# The developmental regulation of CD81 in the rat retina

**Published:** 2007-02-07

**Authors:** Ye Pan, Christina Brown, XiangDi Wang, Eldon E. Geisert

**Affiliations:** 1 Department of Ophthalmology, Hamilton Eye Institute, 930 Madison Avenue, University of Tennessee Health Science Center, Memphis, TN; 2 Undergraduate Student, Department of Biology, Christian Brothers University, 650 East Parkway South; Memphis, TN

## Abstract

**Purpose:**

The tetraspanin CD81 is expressed in Müller glial cells and retinal pigment epithelium (RPE). CD81 and other members of the tetraspanin family link extracellular interactions of cells into intracellular cascades. This study examined the developmental expression of CD81 and protein-protein interactions linking CD81 to intracellular proteins.

**Methods:**

We used synthetic peptides of the C-terminal intracellular domains of CD81 to probe fusion proteins of PDZ domains blotted to nitrocellulose membranes, then confirmed the relationships between the PDZ proteins using immunoprecipitation methods. Colocalization of the associated proteins was analyzed across development, using double-label immunohistochemical methods in the retina of Sprague-Dawley rats.

**Results:**

The C-terminal intracellular sequences of CD81 bound to three putative PDZ domains that potentially represented domains on Sap97 and EBP50. In immunoprecipitation experiments using RPE cells, CD81 coprecipitated with both proteins, EBP50 and Sap97. Like CD81, EBP50 and Sap97 are expressed at low levels immediately after birth and upregulated during the first two postnatal weeks, reaching almost adult levels at postnatal day 20. In the RPE layer, synapse-associated protein 97 (Sap97) and CD81 were associated with the basolateral surface of the cells; ezrin-radixin-moesin-binding phosphoprotein 50 (EBP50) localizing with CD81 was found on microvilli at the inner surface of RPE cells.

**Conclusions:**

These results support the hypothesis that CD81 is associated with the final stages of RPE cell maturation, establishing key molecular interactions linking the cell membrane proteins into macromolecular complexes containing PDZ protein scaffolds.

## Introduction

Previous studies from our laboratory have defined a small membrane protein that regulates glial cell proliferation in response to injury [1] and in brain development [2]. Neither the specific functions nor molecular associations of CD81 are fully understood. In an effort to characterize CD81 fully, we have turned to the developing retina, which offers a unique opportunity to study the expression and developmental interactions of this small membrane protein. CD81 is expressed by Müller glial cells [3] and RPE cells [2]. In rodents, both of these cell types exit the cell cycle after birth [4-6]. Specifically, retinal pigment epithelium (RPE) cells divide up to two weeks, and Müller cells until 14-22 days after birth [5]. Furthermore, both cell types are restricted in the well-characterized laminar structure of the retina, allowing relatively simple anatomical characterization of protein distribution. If CD81 is involved in the final stages of maturation of RPE cells and Müller cells, we can examine its developmental expression pattern relative to that of other proteins co-expressed or associated with CD81.

CD81, like other members of the tetraspanin family of proteins, is a small membrane protein with four transmembrane segments, two small extracellular loops, and small intracellular N and C terminal domains. Tetraspanins form relatively large molecular complexes, called tetraspanin webs, within the plane of the membrane [7-9]. At present, many different membrane proteins can be present within these complexes, including the 12 different mammalian tetraspanins that are known to interact with at least 38 different transmembrane proteins [10-13]. The specific function of the tetraspanin complexes appears to depend on the district proteins forming the complex and the cells in which they are expressed. For example, CD81, through its association with integrin adhesion molecules, can be critically important for cell adhesion and migration [14-19]. These tetraspanin complexes link extracellular events to intracellular signaling cascades [20-24]. When CD81/tetraspanin forms a complex with integrins, it modulates the cells interactions with the extracellular matrix [25-27] through activation of second-messenger systems [23,28,29].

There is a general lack of knowledge about how these proteins link into intracellular processing and second messenger cascades. Examination of the intracellular domains of the tetraspanin family members reveals that CD81 is unique in that it carries a potential PDZ binding domain. At the intracellular C-terminal end of CD81, the sequence SSVY appears; this sequence is similar to a PDZ binding domain [30-32]. Like the tetraspanins, PDZ-containing proteins typically are associated with large complexes of proteins performing localized signaling functions. In the present study, we examined the interactions of CD81 with PDZ proteins in the developing rat retina. Our overall goals were to define the molecular interactions of CD81 with PDZ target proteins and to determine the temporal regulation and distribution of CD81 and PDZ targets in the retina. Since RPE expressed high levels of CD81, we focused on it as well as two PDZ proteins, synapse-associated protein 97 (Sap97) and ezrin-radixin-moesin-binding phosphoprotein 50 (EBP50), which are spatially segregated [33].

## Methods

### Association of CD81 with intracellular proteins

To examine the putative PDZ binding domain on the intracellular, C-terminal end of CD81, we used a modification of a commercially available protocol developed by Panomics (Redwood City, CA). We produced a peptide, H-Gly-Lys-Pro-Ile-Pro-Asn-Pro-Leu-Leu-Gly-Leu-Asp-Ser-Thr- Leu-Cys- Cys-Gly-Ile-Arg-Asn-Ser-Ser-Val-Tyr-OH, containing the C-terminal end of CD81 with its putative PDZ binding domain (in blue color). To allow detection of this peptide in assays, we incorporated the epitope tag for the anti-V5 antibody (red). We also synthesized two control peptides. One had a scrambled amino acid sequence H-Gly-Lys-Pro-Ile-Pro-Asn-Pro-Leu-Leu-Gly-Leu-Asp-Ser-Thr- Tyr-Val- Ser-Ser-Arg-Asn-Ile-Gly-Cys-Cys-Leu-OH; the other contained the C-terminal, intracellular portion of CD81 minus the putative PDZ binding domain H-Gly-Lys-Pro-Ile-Pro-Asn-Pro-Leu-Leu-Gly-Leu-Asp-Ser-Thr- Leu-Cys- Cys-Gly-Ile-Arg-Asn-OH.

We purchased dot blots of glutathione S-transferase (GST) fusion proteins representing all of the known PDZ sequences from Panomics. We probed these blot dots with the experimental peptide and immunostained them with the V5 antibody, which is amouse monoclonal antibody tagged with horseradish peroxidase (HRP; Invitrogen, Carlsbad, CA) then processed them according to the manufacturer's suggested protocol. To monitor chemiluminescence, we placed the blots in the supplied detection buffer and scanned them using a Typhoon 8600 Scanner (Molecular Dynamics). We then analyzed the blots to determine if there were sequence-specific binding to any of the GST fusion proteins, which represented 29 different PDZ domains (Redwood, CA). A total of six Panomic dot blots were run, two for each peptide.

### Antibodies

The antibody directed against rat CD81 was AMP1, developed in our laboratory [1]. Other antibodies were rabbit anti-Sap97 (Affinity BioReagents, Golden, CO), rabbit anti-EBP50 (Affinity BioReagents), mouse anti-CD71, the transferrin receptor (Chemicon, Temecula, CA), and rabbit anti-Ezrin (Santa Cruz Biotechnology, Santa Cruz, CA). Secondary antibodies were either labeled with fluorescent markers or HRP. Peroxidase antibodies used were donkey anti-rabbit or donkey anti-mouse IgG (Jackson ImmunoResearch, West Grove, PA). Fluorescence-labeled secondary antibodies included rhodamine-labeled donkey anti-rabbit (Jackson ImmunoResearch), Alexa 568 goat anti-rabbit (Molecular Probes, Eugene, OR), and Alexa 488 donkey anti-rabbit (Molecular Probes).

### Isolation of proteins associated with CD81 from cultured retinal pigment epithelium

We used Long-Evans rats for tissue-culture experiments to determine the presence of pigment aided in dissections and culturing. RPE cells were cultured using the method of Edwards [34]. In brief, we removed the eyes from rat pups 6 to 8 days of age and placed them in balanced salt solution with 0.3 mM CaCl_2_ for 2-3 h at room temperature in the dark. We placed the globes in 1 mg/ml of trypsin in Hank's balanced salt solution (HBSS) plus 70 units of collagenase (pH 7.8) for 45 min at 37 °C. We then transferred the eyes to medium with 20% fetal calf serum (FCS) and antibiotics and removed the anterior chamber by cutting through the sclera and neural retina just behind the ora serrata. We left the posterior chamber in medium so that the retina expanded, separating from the underlying pigment epithelium. We gently removed the RPE from Bruch's membrane and place it in sheets of RPE in HBSS/EDTA plus 0.1% trypsin for 5 min. We rinsed the disassociated cells and transferred them to a T25 flask. Confluent cultures of rat RPE were rinsed three times in cold 0.01 M phosphate buffered saline (PBS), pH 7.4. We lysed the cells in ice-cold 1% Brij 97 in 0.05 M Tris and 0.05 M NaCl, pH 7.3, containing NaN3 and phenylmethylsulphonyl fluoride (PMSF), then centrifuged at 15,000 x g for 90 min at 4 °C. CD81 and the associated proteins were co-isolated by immunoprecipitation with the AMP1 antibody. We placed the proteins in nonreducing sample buffer, separated them by SDS PAGE, transferred them to nitrocellulose, and blotted with antibodies directed against Sap97, EBP50, and Ezrin.

### Immunoblot analysis

We used one litter of 12 Sprague-Dawley rat pups for the immunoblot analysis. For pups taken before P15, we used hypothermia as anesthesia and decapitated the pups. The mother was anesthetized by intraperitoneal injection of a mixture of xylazine (13 mg/kg, Rompun) and ketamine (87 mg/kg, Ketalar), and her eyes were used as the adult sample. After the animals were sacrificed, their eyes were removed. We dissected the retinas and placed them in nonreducing sample buffer (2% SDS and 10% glycerol in 0.05 M Tris-HCl buffer, pH 6.8). To analyze the concentrations of CD81 in the retina, we used gel electrophoresis and immunoblot methods, similar to those described in Geisert [2]. Using balanced samples of proteins from the retina, we ran them in 4% to 16% acrylamide gels, then transferred them to nitrocellulose. The blots were blocked with 5% nonfat dry milk in borate buffer (pH 8.4) and probed overnight with the primary antibody, AMP1, or anti-EBP50. We then rinsed the blots and probed them with the secondary antibody, HRP-labeled goat anti-mouse. The blots were rinsed with 0.5 M Tris buffer (pH 7.4) and reacted with 0.05% DAB and hydrogen peroxide.

### Immunohistochemistry

We used one litter of 11 postnatal Sprague-Dawley rat pups and the mother in our immunohistochemical analysis of the retina, taking at least two pups at each developmental age: P0, P2, P10, P15, or P20. The mother was used as the adult sample. For the pups taken before P15, we used hypothermia as anesthesia and perfused the pups. The P20 pups and the mother were anesthetized by intraperitoneal injection of a mixture of xylazine (13 mg/kg, Rompun) and ketamine (87 mg/kg, Ketalar) and perfused through the heart with a saline solution followed by 4% paraformaldehyde in 0.1 M phosphate buffer (pH 7.3). After administering the anesthesia, we removed the eyes and placed them in 4% paraformaldehyde overnight. The next day, we transferred the eyes to 30% sucrose for a minimum of 2 days. We cut 16 μm sections of the eyes on a cryostat and placed them on glass slides. The methods used to stain the retina were similar to those previously described [2]. The slides were rinsed in 0.02 M PBS (pH 7.3), placed in 4% BSA containing 0.05% DMSO, and probed overnight with the primary antibody, AMP1 (1:100). After rinsing, we added a donkey anti-mouse secondary antibody (1:200) for 2 h then rinsed the slides in three changes of 0.05 M Tris (pH 7.3). To determine if the CD81 associated with Sap97 or EBP50, we used double-label immunohistochemistry similar to that described in Clarke and Geisert [3]. We first stained the retina with the anti-rat CD81, then added a rabbit antiserum directed against Sap97 or EBP50. The fluorescent-tagged secondary antibodies had been made in donkey and preabsorbed against serum proteins to prevent cross reactivity.

## Results

**Figure 1:**
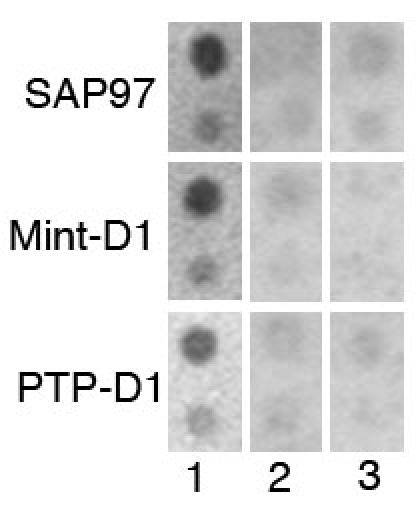
Binding of C-terminal peptides to PDZ domains. The binding of synthetic peptides to dot-blotted GST fusion proteins representing PDZ domains. Lane 1: a peptide that models the C-terminus of CD81 Lane 2: a peptide without the putative PDZ binding domain. Lane 3: the binding of a scrambled protein. Specific binding was observed for the Sap97 domain 3, the protein-tyrosine phosphatase domain 1 (PTP-D1), and the X11L2 protein domain 1 (Mint-D1). Each PDZ domain is spotted with two concentrations of protein: 400 ng (upper spot) and 80 ng (lower spot). GST represents glutathione S-transferase.

The present study began with the observation that CD81 contains a potential PDZ binding domain at its intracellular C-terminus, the amino acid sequence Ser-Ser-Val-Tyr. As a first step in our analysis of the ability of CD81 to interact with PDZ domains, we used synthetic peptides to identify interacting PDZ domains on a dot blot constructed with GST fusion proteins. The first peptide was identical to the C-terminus of CD81; the second was a control peptide missing the putative PDZ binding domain; the third was a control peptide with a scrambled amino-acid sequence. We used the peptides in an overlay assay probing GST-fusion proteins representing different types of PDZ domains. When the blots were analyzed, three different types of PDZ domains bound specifically the C-terminal sequence of CD81 and not to control peptides (Figure 1). One PDZ domain belonged to Sap97. The second and third sets of spots that bound to CD81 peptide represented a PDZ domain type 1. This finding suggested not only that the C-terminal end of CD81 was a PDZ binding domain, but that the C-terminal end bound to a subset of PDZ proteins, Sap-97 and the Domina 1 of Mint and PTP.

The next step was to determine if the proteins expressed by RPE cells contained any of these PDZ domains. We searched our microarray data from RPE cells [35] and consulted the literature [33]. Two candidate proteins were immediately obvious, Sap97 and EBP50, both expressed at high levels in RPE. Sap97 and EBP50 contain all of the types of PDZ domains labeled by the CD81 C-terminal peptide.

**Figure 2:**
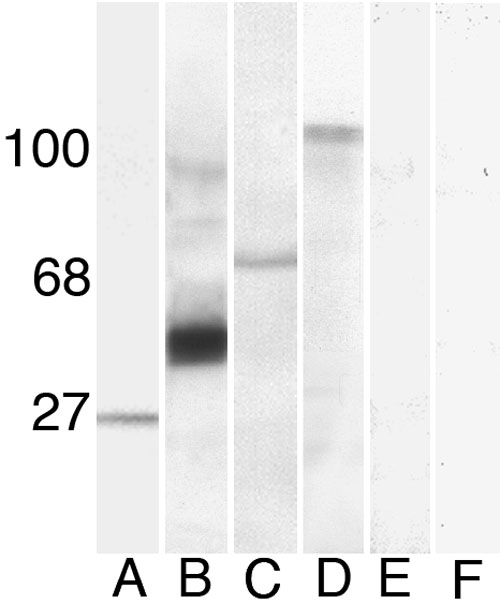
Proteins associated with CD81. Cultured rat retinal pigment epithelium (RPE) cells were solubilized in 1% Brij 97 and immunoprecipitated with mouse anti-rat CD81 antibody. The sample of proteins was then separated by SDS PAGE, transferred to nitrocellulose, and probed with antibodies directed against CD81 (lane A), EBP50 (lane B), Ezrin (lane C), and Sap97 (lane D). Note that CD81 coimmunoprecipitated with EBP50, Ezrin, and Sap97. In control experiments, antibodies directed against the transferrin receptor (CD71) were used to immunoprecipitate proteins from cultured RPE. When these samples were probed for EBP50 (lane E) or Sap97 (lane F), these two PDZ proteins were not observed. Molecular weight markers are indicated to the left in kDa.

To directly test for the binding of CD81 to Sap97 and EBP50, we did a series of immunoprecipitation studies. For these studies, we solubilized cultured rat RPE with a mild detergent, then precipitated complexes of proteins associated with CD81, using antibodies against CD81 attached to Sepharose beads (Figure 2). As expected, a variety of proteins were precipitated along with CD81. To test for both Sap97 and EBP50 among these proteins, we conducted a cross-blotting experiment. We separated the precipitated proteins on SDS PAGE gels, transferred them to nitrocellulose, and probed the blots with antibodies directed against CD81 (Figure 2A), EBP50 (Figure 2B), and Sap97 (Figure 2D). These protein bands were not observed with similar protein samples immunoprecipitated with control mouse IgG (data not shown) or with antibodies against CD71, the transferin receptor (Figure 2E,F). These results demonstrate that CD81 is specifically associated with both Sap97 and EBP50. Since there is a strong association between EBP50 and Ezrin [33,36], we also probed the immunoprecipitated proteins with anti-Ezrin antibodies, observing a strong band (Figure 2C). These results demonstrate a direct link between any tetraspanin (CD81) and an intracellular signaling pathway (Sap97 and EBP50), linking a tetraspanin complex into the cytoskeleton through Ezrin and potentially into second-messenger cascades.

**Figure 3:**
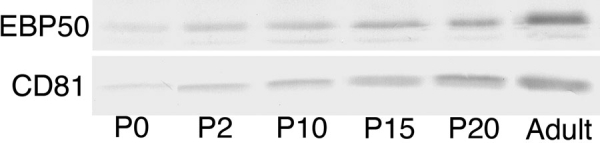
Quantitative changes in CD81 and EBP50. The developmental regulation of EBP50 and CD81 is shown for different developmental ages: postnatal day 0 (P0), P2, P10, P15, P20, and adult. At P0, scant EBP50 and CD81 are present in the retina. There is a gradual increase until P20, when almost adult levels are observed. The levels of these two proteins do not completely mirror each other. EBP50 is expressed only in the microvilli of the retinal pigment epithelium cells, and CD81 is expressed throughout the entire retina.

To further explore the relationship between CD81 and these PDZ proteins, we examined the levels of CD81 and EBP50 in the developing retina, running a series of immunoblots on samples of retina taken at different postnatal time points (Figure 3). At P0, we found a faint but distinct band at 27 kDa, indicating that the levels of CD81 were low. Over the next few days, the levels of CD81 gradually increased, and by P20, CD81 was close to that of the adult level. We then quantified the protein levels in the retina relative to that observed in the adult, using the intensity of the 27 kDa band in three protein samples from individual eyes to determine the relative levels of CD81. We found that the levels of CD81 at P0 were 31% of the adult level. There was increased intensity of the immunopositive band, and at P15, the levels were approximately 85% of that in the adult. We did not measure the levels of Sap97 because its expression is dominated by synaptic formation in the retina relative to the low levels observed in the RPE cell layer. We did measure the levels of EBP50 because this protein is almost exclusively expressed in the RPE cells (Figure 3). We found, as with CD81, that the levels of EBP50 were low at P0 but high by P20. Thus, in addition to the dramatic upregulation of CD81 during postnatal development of the retina, at least one PDZ, EBP50, follows the same pattern.

**Figure 4:**
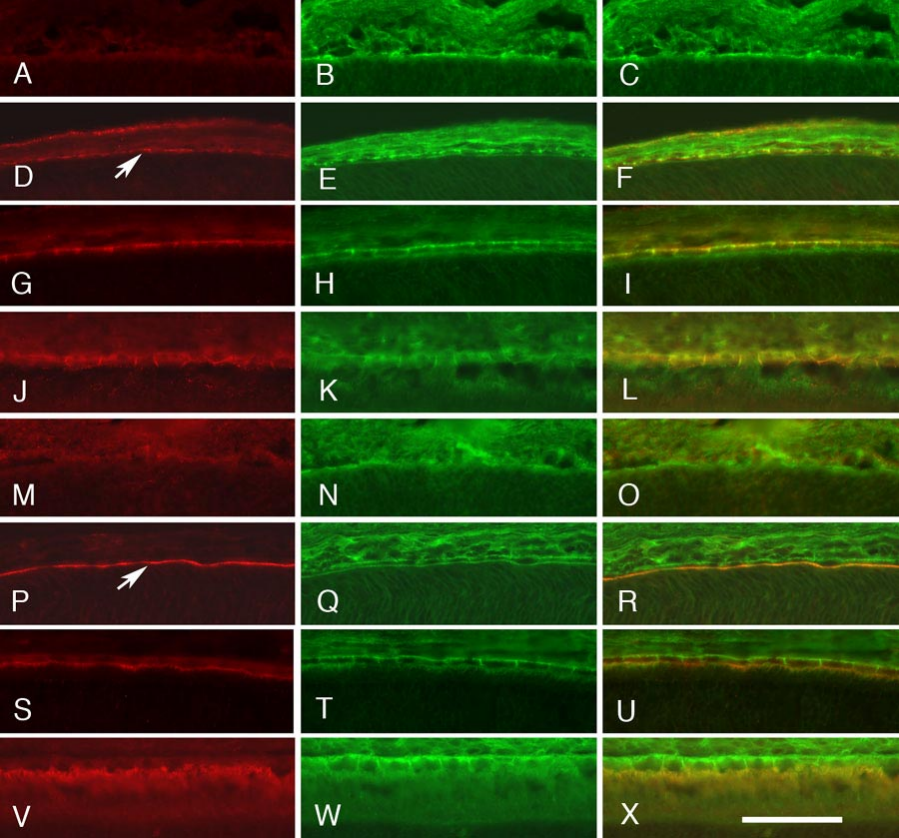
Colocalization of CD81 with Sap97 and EBP50 in the developing retinal pigment epithelium layer. The developmental pattern of expression of Sap97 (**A**, **D**, **G**, **J**), EBP50 (**M**, **P**, **S**, **V**), and CD81 (**B**, **E**, **H**, **K**, **N**, **Q**, **T**, **W**) is shown for the retinal pigment epithelium (RPE) cell layer in double-stained sections at different developmental ages: P0 (**A**-**C**, **M**-**O**), P2 (**D**-**F**, **P**-**R**), P10 (**G**-**I**, **S**-**U**), and P15 (**J**-**L**, **V**-**X**). The merged images are shown to the right (**C**, **F**, **I**, **L**, **O**, **R**, **U**, **X**). At P0 (**A**-**C**, **M**-**O**) CD81 levels are low, with only a faint outline of the developing RPE. There is little immunostaining for Sap97 (**A**) or EBP50 (**M**). By P2 (**D**-**F**, **P**-**R**), CD81 defined the cellular membranes of the RPE cells. There is a clear upregulation of Sap97 along the basal surface of the RPE (**D**, arrow). Sap97 is distributed on the basolateral surfaces of the RPE cells (**F**). The levels of EBP50 are also increasing (**P**), forming a tight band of immunoreactivity at the junction between the RPE cells and the developing photoreceptors (arrow). EBP50 immunoreactivity labels the apical surface of RPE cells (**R**). The levels of all three proteins increase over time, and the immunolabeling pattern becomes more distinct at P10 and P15. All photomicrographs were taken at the same magnification. In the scale bar, X=25 μm.

**Figure 5:**
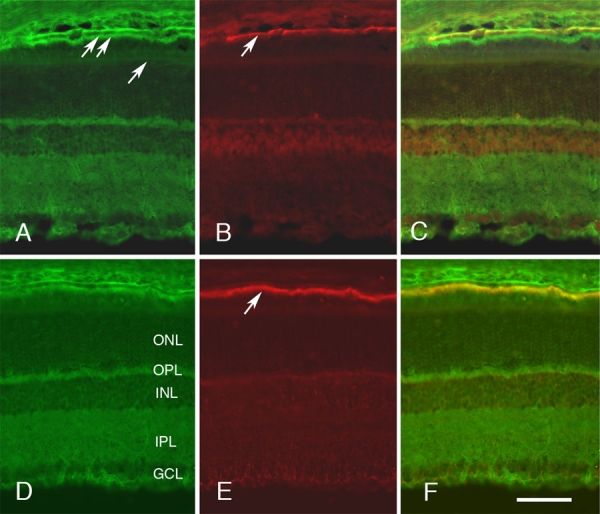
Colocalization of CD81 with Sap97 and EBP50 at P20. The staining of the P20 rat retina for CD81 (**A** and **D**), Sap97 (**B**), and EBP50 (**E**) is shown in two double-stained sections. The merged images of these two sections are shown in **C** and **F**. The pattern of CD81 labeling is consistent with the labeling of Müller glial cells, as shown by the prominent labeling of the external limiting membrane (arrow, **A**) and retinal pigment epithelium (RPE) cells (double arrow, **A**). Sap97 shows a general stain in the retinal neurons and a prominent band at the base of the RPE (arrow in **B**). In the merged image (**C**), Sap97 immunoreactivity is colocalized with CD81 on the basolateral surface of RPE cells. EBP50 labels a prominent band at the junction between the retina and the PRE cells (arrow, **E**). The EBP50 immunoreactivity colocalizes with CD81 immunoreactivity at the apical surface of the RPE cells (**F**). In **A** and **F**, the layers of the P20 retina are shown: the ganglion cell layer (GCL), inner plexiform layer (IPL), inner nuclear layer (INL), outer plexiform layer (OPL), and outer nuclear layer (ONL). All photomicrographs are taken at the same magnification. The scale bar in **F** represents 25 μm.

We extended this analysis by looking at the change in the developmentally regulated immunolabeling pattern in the RPE cell layer at closely spaced postnatal developmental time points (Figure 4). In the developing retina, the RPE layer is adjacent to the outer edge of the retina next to future photoreceptor layer. At P0 (Figure 4A-C, Figure 4M-O), there is little immunolabeling for Sap97 (Figure 4A), CD81 (Figure 4B,N), or EBP50 (Figure 4M). By P2, all proteins begin to increase in expression and reveal a distinctive pattern of labeling, with Sap97 at the basal surface of the developing RPE cells (Figure 4D, arrow) and EBP50 at the dorsal inner surface of these cells (Figure 4P, arrow). The labeling on the inner surface appears to mark the emerging microvilli on the RPE that eventually will surround the outer segments of the photoreceptors. This pattern continues to emerge until by P20 the adult pattern is observed. For Sap97, the antibody is localized to the dorsal lateral and ventral surfaces of the RPE cells (Figure 5B). EBP50 is diametrically apposed, selectively labeling the microvilli on the inner surface of the RPE cells (Figure 5E). Both of these intracellular proteins appear to be colocalized with CD81, forming two distinct molecular groupings: one on the basolateral surface of the RPE cells (Figure 5C) and the other on the inner surface of the RPE cells (Figure 5F).

**Figure 6:**
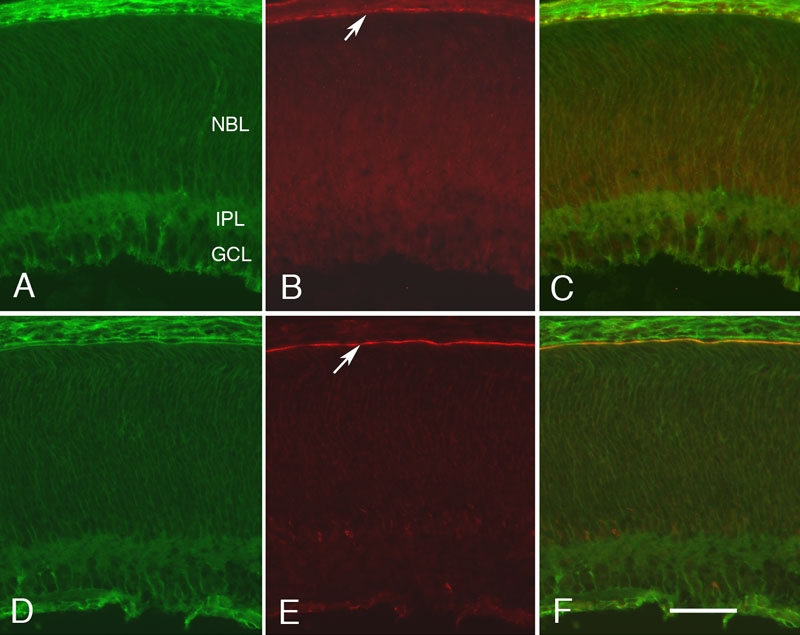
Colocalization of CD81 with Sap97 and EBP50 at P2. The staining pattern of CD81, Sap97, and EBP50 in the developing rat retina is shown at P2. **A** and **D** are stained for CD81, **B** is stained for Sap97, and **C** is the merged image. **E** is stained for EBP50; **F** is the merged image. At this age, the ganglion cell layer (GCL) and inner plexiform layer (IPL) are present. The remainder of the retina consists of a neuroblastic layer (NBL). The arrows in **B** and **E** indicate the location of the developing retinal pigment epithelium (RPE) layer. All photomicrographs were taken at the same magnification. The scale bar in represents 25 μm.

We next focused on the general pattern of CD81, EBP50 and Sap97, at two time points, P2 (Figure 6) and P20 (Figure 5). The first distinct pattern of labeling in the retina is seen at P2, when there was light generalized labeling for CD81 (Figure 6A,D). The ganglion layer had the highest levels of CD81 immunoreactivity, which outlined groups of cell bodies. The next highest level of CD81 labeling was in the inner plexiform layer with light labeling around the undifferentiated neuroblasts that make up the majority of retinal cells in the P2 retina. In addition, there was relatively prominent labeling of the developing blood vessels in the inner retina. The pattern observed at P2 provides the first indication of specific labeling by Sap97and EBP50. Sap97 labeling appeared in the developing RPE cell layer (Figure 6B) and was strongly associated with CD81 at the base of these cells (Figure 6C). In the developing retina, EBP50 labeled a prominent band in the developing retina (Figure 6E) that is associated with the inner surface of the developing RPE cell layer (Figure 6F).

The pattern of labeling at P20 was similar to that observed in the adult retina and was suggestive of labeling of Müller cells and astrocytes (Figure 5A,D). There was prominent labeling of the retina from the internal limiting membrane to the outer limiting membrane. In addition, the inner limiting membrane was clearly labeled. This pattern is similar to that which we previously described (Clarke and Geisert 1998) [3]. The prominent labeling of the external limiting membrane and increased immunoreactivity around blood vessels, especially in the ganglion cell layer, was indicative of labeling of Müller glial cells and astrocytes in the ganglion cell layer.

## Discussion

Previous study of the developing brain and spinal cord [14] demonstrated that CD81 is upregulated at approximately the same time that glial cells exit the cell cycle. A similar temporal pattern of expression was observed in the developing retina. CD81 is expressed by Müller glial cells [3] and RPE cells [2]. In the postnatal retina [4,5], cells of the RPE actively divide through P7 [6]; Müller cells proliferate up to P22 [5]. During the end of mitotic activity, we observed a significant increase in the levels of CD81 as well as patterns of expression consistent with CD81 being expressed by RPE cells and Müller cells. The fact that CD81 is upregulated at the specific time these cells stop dividing indicates that this protein has a critical function in the final stages of development, maturation, and normal function of the adult retina.

This study has allowed us to examine CD81 and its potential intracellular interactions in RPE cells, for these cells develop postnatally and represent a well-characterized epithelium. In general, CD81 and other members of the tetraspanin family participate in a wide variety of molecular complexes. These associations of CD81 with different membrane proteins result in molecular complexes, representing diverse microdomains within the cell membrane. CD81 forms a complex with CD19 and CD21 in the mnemonic response of B cells [9,24,37]. In addition, CD81 is critical for cell adhesion and migration through its association with adhesion molecules [14-19]. In both cases, the tetraspanin complexes link extracellular events to intracellular signaling cascades [20-24]. These links are revealed by antibody binding to CD81 that activates a tyrosine kinase in human lymphocytes [22,23,28].

By partnering with different membrane proteins, CD81 is involved in a variety of different cellular functions [15,18,22,38,39]. For example, when CD81 forms a complex with integrins, it is directly involved in regulating cellular interactions with the extracellular matrix [25-27]. This interaction has a defined effect on second-messenger systems [23,28,29] and, eventually, modulates the mitotic activity of the cell [1,40,41].

Given all that we know about tetraspanin interactions with membrane proteins, there remains a distinct lack of information about the linkage of the tetraspanin complexes into intracellular compartments. Recently, Sala-Valdes et al. [13] demonstrated linkage of CD9 directly to Ezrin protein. This interaction links EWI-2 and EWI-F proteins, along with CD81, directly to actin through the CD9/Exrin complex to the actin cytoskeleton. Like the complex observed by Sala-Valdes et al. [13], CD81 in the RPE cells is associated with E EWI-2, EWI-F, CD9, b1 integrin, a3 integrins, and a5 integrin [42]. Furthermore, the RPE cells allows us one of the first glimpses into the potential linkage of tetraspanin complexes through CD81 into intracellular components.

We found that the amino acid sequence on the C-terminal end of CD81 appeared to have the ability to bind a PDZ binding domain. When we tested synthetic peptides of the C-terminal domain against GST fusion proteins, we observed that the peptide specifically bound to three spots on the Panomic dot blot. The Sap97 spots represent the PDZ domain 3 of Sap97. Examination of microarray data from RPE cells [35] revealed that the most likely candidates were Sap97 and EBP50. EBP50 has two PDZ domains, including PDZ domain 1 [36]. Immunoprecipitation studies confirmed an association among CD81, Sap97, and EBP50.

In adult rats, we found CD81 on all surfaces of the RPE cells [2], while EBP50 was associated with microvilli on the inner surface and Sap97 was on the basolateral surface of the cells [33]. Upregulation of all three proteins followed approximately the same time course. As RPE cells mature, these proteins occupy their specific subcellular localizations and, presumably, carry out their adult functions. Due to the distinct locations of these two PDZ proteins within the RPE, CD81 must be involved in at least two different molecular complexes, one in the microvilli associated with EBP50/Ezrin and the other associated with Sap97 on the lateral and basal cell surfaces. Indeed, this is what we found. On the apical surface of the cells, CD81 is associated with EBP50, and these proteins are upregulated on a similar time course as the RPE matures.

What are the specific functional roles of each of the CD81/tetraspanin complexes? At present, we do not know the specific molecular components of each complex. However, we do know that CD81 is associated with EBP50 on the inner RPE microvilli. Recent studies [43,44] have found that EBP50 is associated with cellular retinaldehyde-binding protein (CRALBP). This molecular interaction localizes CRALBP to the precise subcellular compartment where the shed outer segments of rods are ingested by the RPE and where retinoid processing occurs. It is tempting to speculate that the PDZ protein EBP50 links the CD81 complex in the microvilli to CRALBP. If the CD81/EBP50 complex is associated with the ingestion of shed outer rod segments, this association through EBP50 would bring the molecular machinery for retinoid processing into direct association with the shed outer rod segments.

We also found CD81 colocalized with Sap97 on the basolateral surface. Immunohistochemical examination showed a similar upregulation during early postnatal development. One could speculate that CD81 associated with Sap97 on the basolateral surface of cells has an important function in the final stages of RPE cell development and the stabilization of cellular contacts. In the postnatal retina, RPE cells and Müller cells continue to divide until the appropriate number of cells is reached. In the final stages of retinal development, these two cell types downregulate their cell cycles and enter G0 [4,6]. The stopping of proliferation and the attainment of final maturation are developmental consequences of differentiation. After injury of the adult retina, these two cell types can reenter S phase and proliferate again [45,46], frequently resulting in the formation of cellular membranes termed proliferative vitreoretinopathy [47-50]. These membranes can contract, causing the retina to detach.

In our effort to understand the molecular mechanism governing the regulation of glial proliferation and reactivity, we focused on a member of the tetraspanin family of proteins, CD81. This tetraspanin was originally termed the target of an antiproliferative antibody [40], since antibodies directed against CD81 depressed the mitotic activity of cultured cells. A considerable amount of experimental findings indicate that CD81 is part of one of the molecular mechanisms regulating the cell cycle as well as cell migration [9,11,41]. When the normal stabilizing functions of CD81 are disrupted, as in photoreceptor cell death, the complex may signal to the cell to begin migrating and dividing again. We currently are pursuing further studies of the function of CD81 following injury.

## References

[1] Geisert EE, Yang L, Irwin MH (1996). Astrocyte growth, reactivity, and the target of the antiproliferative antibody, TAPA.. J Neurosci.

[2] Geisert EE, Abel HJ, Fan L, Geisert GR (2002). Retinal pigment epithelium of the rat express CD81, the target of the anti-proliferative antibody (TAPA).. Invest Ophthalmol Vis Sci.

[3] Clarke K, Geisert EE (1998). The target of the antiproliferative antibody (TAPA) in the normal and injured rat retina.. Mol Vis.

[4] Young RW (1985). Cell differentiation in the retina of the mouse.. Anat Rec.

[5] Stroeva OG, Panova IG (1983). Retinal pigment epithelium: pattern of proliferative activity and its regulation by intraocular pressure in postnatal rats.. J Embryol Exp Morphol.

[6] Bodenstein L, Sidman RL (1987). Growth and development of the mouse retinal pigment epithelium. I. Cell and tissue morphometrics and topography of mitotic activity.. Dev Biol.

[7] Le Naour F, Charrin S, Labas V, Le Caer JP, Boucheix C, Rubinstein E (2004). Tetraspanins connect several types of Ig proteins: IgM is a novel component of the tetraspanin web on B-lymphoid cells.. Cancer Immunol Immunother.

[8] Levy S, Shoham T (2005). Protein-protein interactions in the tetraspanin web.. Physiology (Bethesda).

[9] Shoham T, Rajapaksa R, Kuo CC, Haimovich J, Levy S (2006). Building of the tetraspanin web: distinct structural domains of CD81 function in different cellular compartments.. Mol Cell Biol.

[10] Hemler ME (2003). Tetraspanin proteins mediate cellular penetration, invasion, and fusion events and define a novel type of membrane microdomain.. Annu Rev Cell Dev Biol.

[11] Boucheix C, Rubinstein E (2001). Tetraspanins.. Cell Mol Life Sci.

[12] Berditchevski F (2001). Complexes of tetraspanins with integrins: more than meets the eye.. J Cell Sci.

[13] Sala-Valdes M, Ursa A, Charrin S, Rubinstein E, Hemler ME, Sanchez-Madrid F, Yanez-Mo M (2006). EWI-2 and EWI-F link the tetraspanin web to the actin cytoskeleton through their direct association with ezrin-radixin-moesin proteins.. J Biol Chem.

[14] Ikeyama S, Koyama M, Yamaoko M, Sasada R, Miyake M (1993). Suppression of cell motility and metastasis by transfection with human motility-related protein (MRP-1/CD9) DNA.. J Exp Med.

[15] Yanez-Mo M, Alfranca A, Cabanas C, Marazuela M, Tejedor R, Ursa MA, Ashman LK, de Landazuri MO, Sanchez-Madrid F (1998). Regulation of endothelial cell motility by complexes of tetraspan molecules CD81/TAPA-1 and CD151/PETA-3 with alpha3 beta1 integrin localized at endothelial lateral junctions.. J Cell Biol.

[16] Hemler ME (1998). Integrin associated proteins.. Curr Opin Cell Biol.

[17] Fitter S, Sincock PM, Jolliffe CN, Ashman LK (1999). Transmembrane 4 superfamily protein CD151 (PETA-3) associates with beta 1 and alpha IIb beta 3 integrins in haemopoietic cell lines and modulates cell-cell adhesion.. Biochem J.

[18] Stipp CS, Kolesnikova TV, Hemler ME (2001). EWI-2 is a major CD9 and CD81 partner and member of a novel Ig protein subfamily.. J Biol Chem.

[19] Charrin S, Le Naour F, Oualid M, Billard M, Faure G, Hanash SM, Boucheix C, Rubinstein E (2001). The major CD9 and CD81 molecular partner. Identification and characterization of the complexes.. J Biol Chem.

[20] Jennings LK, Fox CF, Kouns WC, McKay CP, Ballou LR, Schultz HE (1990). The activation of human platelets mediated by anti-human platelet p24/CD9 monoclonal antibodies.. J Biol Chem.

[21] Yatomi Y, Ozaki Y, Satoh K, Kume S (1993). Anti-CD9 monoclonal antibody elicits staurosporine inhibitable phosphatidylinositol 4,5-bisphosphate hydrolysis, phosphatidylinositol 3,4-bisphosphate synthesis, and protein-tyrosine phosphorylation in human platelets.. FEBS Lett.

[22] Schick MR, Nguyen VQ, Levy S (1993). Anti-TAPA-1 antibodies induce protein tyrosine phosphorylation that is prevented by increasing intracellular thiol levels.. J Immunol.

[23] Berditchevski F, Tolias KF, Wong K, Carpenter CL, Hemler ME (1997). A novel link between integrins, transmembrane-4 superfamily proteins (CD63 and CD81), and phosphatidylinositol 4-kinase.. J Biol Chem.

[24] Carloni V, Mazzocca A, Ravichandran KS (2004). Tetraspanin CD81 is linked to ERK/MAPKinase signaling by Shc in liver tumor cells.. Oncogene.

[25] Mannion BA, Berditchevski F, Kraeft SK, Chen LB, Hemler ME (1996). Transmembrane-4 superfamily proteins CD81 (TAPA-1), CD82, CD63, and CD53 specifically associated with integrin alpha 4 beta 1 (CD49d/CD29).. J Immunol.

[26] Levy S, Todd SC, Maecker HT (1998). CD81 (TAPA-1): a molecule involved in signal transduction and cell adhesion in the immune system.. Annu Rev Immunol.

[27] Stipp CS, Hemler ME (2000). Transmembrane-4-superfamily proteins CD151 and CD81 associate with alpha 3 beta 1 integrin, and selectively contribute to alpha 3 beta 1-dependent neurite outgrowth.. J Cell Sci.

[28] Yauch RL, Hemler ME (2000). Specific interactions among transmembrane 4 superfamily (TM4SF) proteins and phosphoinositide 4-kinase.. Biochem J.

[29] Zhang XA, Bontrager AL, Hemler ME (2001). Transmembrane-4 superfamily proteins associate with activated protein kinase C (PKC) and link PKC to specific beta(1) integrins.. J Biol Chem.

[30] Sheng M, Sala C (2001). PDZ domains and the organization of supramolecular complexes.. Annu Rev Neurosci.

[31] Denker BM, Nigam SK (1998). Molecular structure and assembly of the tight junction.. Am J Physiol.

[32] Garner CC, Nash J, Huganir RL (2000). PDZ domains in synapse assembly and signalling.. Trends Cell Biol.

[33] Bonilha VL, Rodriguez-Boulan E (2001). Polarity and developmental regulation of two PDZ proteins in the retinal pigment epithelium.. Invest Ophthalmol Vis Sci.

[34] Edwards RB (1981). The isolation and culturing of retinal pigment epithelium of the rat.. Vision Res.

[35] Rogojina AT, Orr WE, Song BK, Geisert EE (2003). Comparing the use of Affymetrix to spotted oligonucleotide microarrays using two retinal pigment epithelium cell lines.. Mol Vis.

[36] Short DB, Trotter KW, Reczek D, Kreda SM, Bretscher A, Boucher RC, Stutts MJ, Milgram SL (1998). An apical PDZ protein anchors the cystic fibrosis transmembrane conductance regulator to the cytoskeleton.. J Biol Chem.

[37] Cherukuri A, Shoham T, Sohn HW, Levy S, Brooks S, Carter R, Pierce SK (2004). The tetraspanin CD81 is necessary for partitioning of coligated CD19/CD21-B cell antigen receptor complexes into signaling-active lipid rafts.. J Immunol.

[38] Takahashi S, Doss C, Levy S, Levy R (1990). TAPA-1, the target of an antiproliferative antibody, is associated on the cell surface with the Leu-13 antigen.. J Immunol.

[39] Bradbury LE, Kansas GS, Levy S, Evans RL, Tedder TF (1992). The CD19/CD21 signal transducing complex of human B lymphocytes includes the target of antiproliferative antibody-1 and Leu-13 molecules.. J Immunol.

[40] Oren R, Takahashi S, Doss C, Levy R, Levy S (1990). TAPA-1, the target of an antiproliferative antibody, defines a new family of transmembrane proteins.. Mol Cell Biol.

[41] Hemler ME (2001). Specific tetraspanin functions.. J Cell Biol.

[42] Geisert EE, Wang XD, Pan Y. Interactions of CD81 with intracellular and extracellular proteins in RPE cells.ARVO Annual Meeting: 2005 Fort Lauderdale (FL).

[43] Nawrot M, West K, Huang J, Possin DE, Bretscher A, Crabb JW, Saari JC (2004). Cellular retinaldehyde-binding protein interacts with ERM-binding phosphoprotein 50 in retinal pigment epithelium.. Invest Ophthalmol Vis Sci.

[44] Nawrot M, Liu T, Garwin GG, Crabb JW, Saari JC (2006). Scaffold Proteins and Visual Pigment Regeneration.. Photochem Photobiol.

[45] Dyer MA, Cepko CL (2000). Control of Muller glial cell proliferation and activation following retinal injury.. Nat Neurosci.

[46] Hinton DR, He S, Jin ML, Barron E, Ryan SJ (2002). Novel growth factors involved in the pathogenesis of proliferative vitreoretinopathy.. Eye.

[47] Charteris DG (1995). Proliferative vitreoretinopathy: pathobiology, surgical management, and adjunctive treatment.. Br J Ophthalmol.

[48] Campochiaro PA (1997). Pathogenic mechanisms in proliferative vitreoretinopathy.. Arch Ophthalmol.

[49] Hiscott P, Sheridan C, Magee RM, Grierson I (1999). Matrix and the retinal pigment epithelium in proliferative retinal disease.. Prog Retin Eye Res.

[50] Fisher SK, Erickson PA, Lewis GP, Anderson DH (1991). Intraretinal proliferation induced by retinal detachment.. Invest Ophthalmol Vis Sci.

